# Surveillance for Coccidioidomycosis — United States, 2011–2017

**DOI:** 10.15585/mmwr.ss6807a1

**Published:** 2019-09-20

**Authors:** Kaitlin Benedict, Orion Z. McCotter, Shane Brady, Kenneth Komatsu, Gail L. Sondermeyer Cooksey, Alyssa Nguyen, Seema Jain, Duc J. Vugia, Brendan R. Jackson

**Affiliations:** ^1^Division of Foodborne, Waterborne, and Environmental Diseases, National Center for Emerging and Zoonotic Infectious Diseases, CDC, Atlanta, Georgia; ^2^Arizona Department of Health Services, Phoenix, Arizona; ^3^Division of Communicable Disease Control, California Department of Public Health, Richmond, California; ^4^Division of Communicable Disease Control, California Department of Public Health, Sacramento, California

## Abstract

**Problem/Condition:**

Coccidioidomycosis (Valley fever) is an infection caused by the environmental fungus *Coccidioides* spp., which typically causes respiratory illness but also can lead to disseminated disease. This fungus typically lives in soils in warm, arid regions, including the southwestern United States.

**Reporting Period:**

2011–2017.

**Description of System:**

Coccidioidomycosis has been nationally notifiable since 1995 and is reportable in 26 states and the District of Columbia (DC), where laboratories and physicians notify local and state public health departments about possible coccidioidomycosis cases. Health department staff determine which cases qualify as confirmed cases according to the definition established by Council of State and Territorial Epidemiologists and voluntarily submit basic case information to CDC through the National Notifiable Diseases Surveillance System.

**Results:**

During 2011–2017, a total of 95,371 coccidioidomycosis cases from 26 states and DC were reported to CDC. The number of cases decreased from 2011 (22,634 cases) to 2014 (8,232 cases) and subsequently increased to 14,364 cases in 2017; >95% of cases were reported from Arizona and California. Reported incidence in Arizona decreased from 261 per 100,000 persons in 2011 to 101 in 2017, whereas California incidence increased from 15.7 to 18.2, and other state incidence rates stayed relatively constant. Patient demographic characteristics were largely consistent with previous years, with an overall predominance among males and among adults aged >60 years in Arizona and adults aged 40–59 years in California.

**Interpretation:**

Coccidioidomycosis remains an important national public health problem with a well-established geographic focus. The reasons for the changing trends in reported cases are unclear but might include environmental factors (e.g., temperature and precipitation), surveillance artifacts, land use changes, and changes in the population at risk for the infection.

**Public Health Action:**

Health care providers should consider a diagnosis of coccidioidomycosis in patients who live or work in or have traveled to areas with known geographic risk for *Coccidioides* and be aware that those areas might be broader than previously recognized. Coccidioidomycosis surveillance provides important information about the epidemiology of the disease but is incomplete both in terms of geographic coverage and data availability. Expanding surveillance to additional states could help identify emerging areas that pose a risk for locally acquired infections. In Arizona and California, where most cases occur, collecting systematic enhanced data, such as more detailed patient characteristics and disease severity, could help clarify the reasons behind the recent changes in incidence and identify additional opportunities for focused prevention and educational efforts.

## Introduction

Coccidioidomycosis (Valley fever) is an infection caused by the environmental fungus *Coccidioides* spp. Approximately 40% of infected persons develop symptoms including fatigue, cough, fever, shortness of breath, and headache, typically after a 1- to 3-week incubation period (*1*). The infection is often clinically indistinguishable from community-acquired pneumonia caused by other pathogens, which can lead to inappropriate treatment, including antibacterial agents (*2*,*3*). A small proportion of patients develop life-threatening severe pulmonary disease or disseminated disease that can lead to chronic sequelae requiring lifelong treatment; known risk factors include immunosuppression, black race, and Filipino ethnicity (*1*).

The geographic distribution of *Coccidioides* includes the southwestern United States and parts of Mexico and Central and South America. Arizona’s Sonoran Desert, which includes the metropolitan areas of Phoenix and Tucson, and California’s southern San Joaquin Valley are particularly high-risk areas. *Coccidioides* is also known to be present in Nevada, New Mexico, Utah, and Texas, although to a lesser extent (*4*). The fungus also was recently discovered in south-central Washington, suggesting that its true range is likely broader than recognized (*5*). However, most cases acquired outside of the southwestern United States are travel associated and have been associated with long diagnostic delays, likely because clinicians in areas to which travelers are returning are unfamiliar with the disease (*6*). Rare fomite-transmitted cases (e.g., on personal items such as a suitcase or shoes and on agricultural products including cotton bales) also have been documented (*7*).

Coccidioidomycosis has been nationally notifiable since 1995. Previous reports describe a significant nationwide increase in reported cases during 1998–2011, in California during 2016–2017, and in Arizona during 2017–2018 (*8*–*10*). This summary provides an update on the epidemiology of coccidioidomycosis during 2011–2017 using national surveillance data. This report is intended for public health officials, health care providers, and stakeholders in the health care industry to promote awareness of the incidence, demographic patient characteristics, and geographic distribution and trends of coccidioidomycosis.

## Methods

### Data Source

State health departments voluntarily send information on coccidioidomycosis cases to the National Notifiable Diseases Surveillance System (NNDSS) (https://wwwn.cdc.gov/nndss). During 2011–2017, coccidioidomycosis was reportable in 18–24 states per year. A few states also transmitted information on coccidioidomycosis cases even though the condition was not officially reportable in those jurisdictions; these cases were excluded from this analysis. Variables included age, sex, race/ethnicity, primary state and county of residence, and event date. Event date represented the earliest date associated with the case, which could be the symptom onset date, diagnosis date, laboratory test date, or the date reported to the county or state health department. Although some public health jurisdictions routinely collected additional data such as travel history and outcomes, this information was not available in NNDSS.

### Surveillance Case Definition

Laboratories and health care providers send reports of potential coccidioidomycosis cases to state and local health departments, which use the case definition established by the Council of State and Territorial Epidemiologists (CSTE) to determine whether cases meet the criteria for a confirmed case. The 2011 CSTE definition includes both laboratory and clinical criteria (*11*). The laboratory criteria include cultural, histopathologic, or molecular evidence of the presence of *Coccidioides* species; a positive serologic test (e.g., enzyme immunoassay, immunodiffusion, and complement fixation) for coccidioidal immunoglobulin M (IgM) or immunoglobulin G (IgG) antibodies in serum, cerebrospinal fluid, or other body fluid; or coccidioidal skin test conversion from negative to positive. Most cases are diagnosed with serologic testing (*12*). The CSTE clinical criteria broadly refer to an influenza-like or pneumonia-like febrile illness or dissemination to multiple organ systems and also state that the infection might be asymptomatic, leading to ambiguity about whether clinical manifestations are required to meet the case definition. Since 1997, Arizona has used only the laboratory component of the CSTE case definition to designate coccidioidomycosis cases as confirmed because of the state’s numerous cases and the resource-intensive nature of investigating clinical signs and symptoms (*13*). Enhanced surveillance in Arizona during 2007–2008 indicated that 5% of persons with reported cases had no symptoms or had symptoms inconsistent with the CSTE case definition, suggesting that a laboratory-only definition was sufficiently specific for surveillance in Arizona (*14*). As of January 2019, California also no longer requires clinical confirmation of disease, although some counties had previously been using a laboratory-only definition (*15*). States other than Arizona and California generally use both the laboratory and the clinical components of the CSTE definition to classify cases as confirmed (*6*). Certain states classify cases as probable or suspect, typically when they are unable to obtain clinical information, even though these classifications do not exist in the CSTE case definition.

### Analysis

This analysis included confirmed cases reported to CDC during 2011–2017 from all states where coccidioidomycosis was reportable in a given year (and cases with unknown status from California during 2011–2012). Overall, county-, age-, sex-, and race/ethnicity-specific annual incidences per 100,000 population were calculated using intercensal estimates from the U.S. Census Bureau. To compare characteristics by area, demographic characteristics, case counts, and incidence were assessed for Arizona; California; Nevada, New Mexico, and Utah combined; and all other states where coccidioidomycosis was reportable combined. These analytic groupings were chosen because Arizona and California report most cases; *Coccidioides* is also known to be present in Nevada, New Mexico, and Utah, although they report fewer cases than Arizona and California; and cases reported from other states are typically travel associated (*8*). Negative binomial regression was performed to assess trends in coccidioidomycosis incidence, and α = 0.05 was considered statistically significant. To avoid potentially unreliable estimates, the average annual percent change was not calculated for counties with <25 cases reported during the study period. All analyses were conducted using SAS software (version 9.4; SAS Institute).

## Results

**FIGURE 1:**
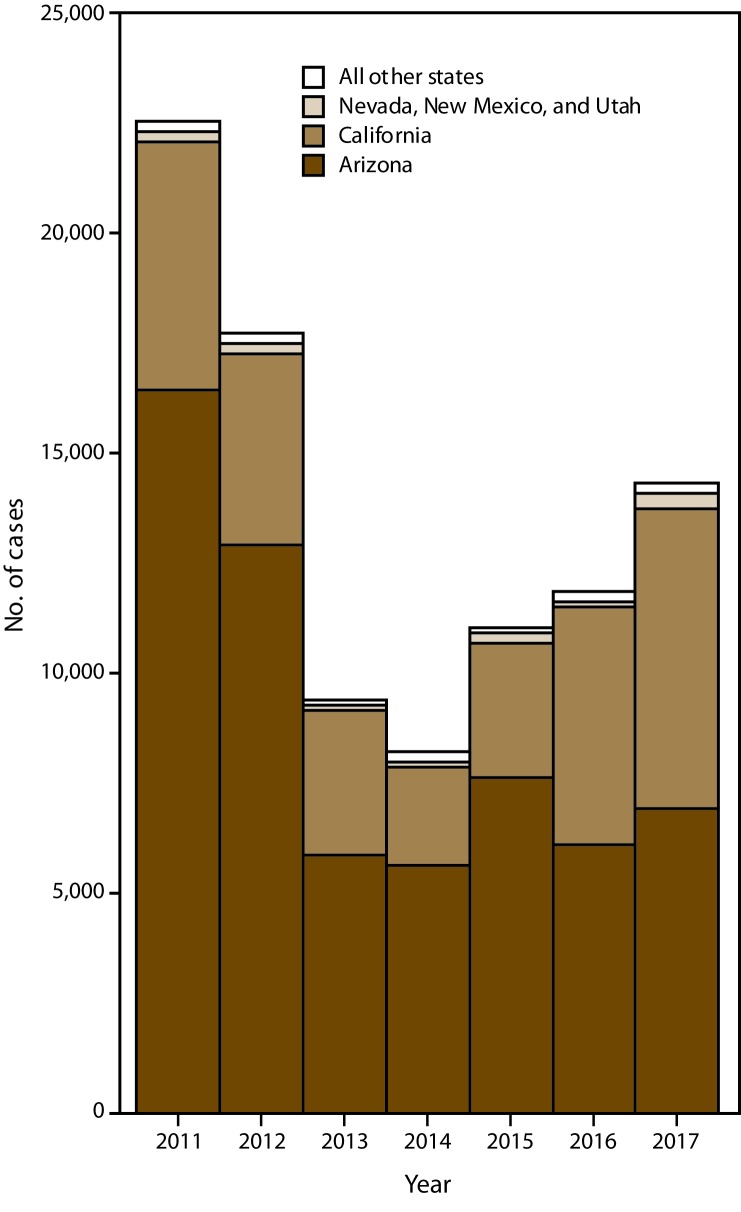
Annual number of coccidioidomycosis cases, by area* — 26 states and the District of Columbia, 2011–2017 * All other states refers to all other states where coccidioidomycosis was reportable.

During 2011–2017, a total of 95,371 coccidioidomycosis cases were reported to CDC from 26 states (Arizona, Arkansas, California, Delaware, Indiana, Kentucky, Louisiana, Maryland, Michigan, Minnesota, Missouri, Montana, Nebraska, Nevada, New Hampshire, New Mexico, North Dakota, Ohio, Oregon, Pennsylvania, Rhode Island, South Dakota, Utah, Washington, Wisconsin, and Wyoming) and the District of Columbia (DC) (Figure 1). Most cases were reported from Arizona (61,480 [64.5%]) and California (30,979 [32.5%]). Fewer cases were reported from Nevada, New Mexico, and Utah combined (1,394 [1.5%]) and all other states combined where coccidioidomycosis was reportable (1,518 [1.6%]).

**FIGURE 2:**
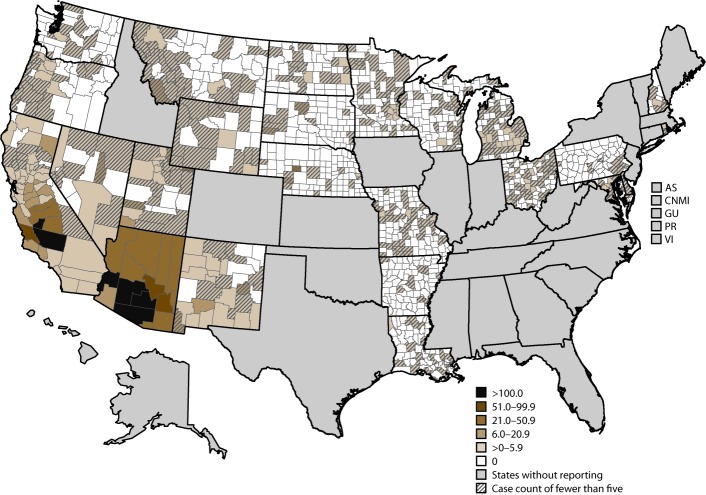
Average annual incidence* of coccidioidomycosis, by county — 26 states and the District of Columbia, 2011–2017 * Per 100,000 population, calculated using intercensal estimates from the U.S. Census Bureau.

**FIGURE 3:**
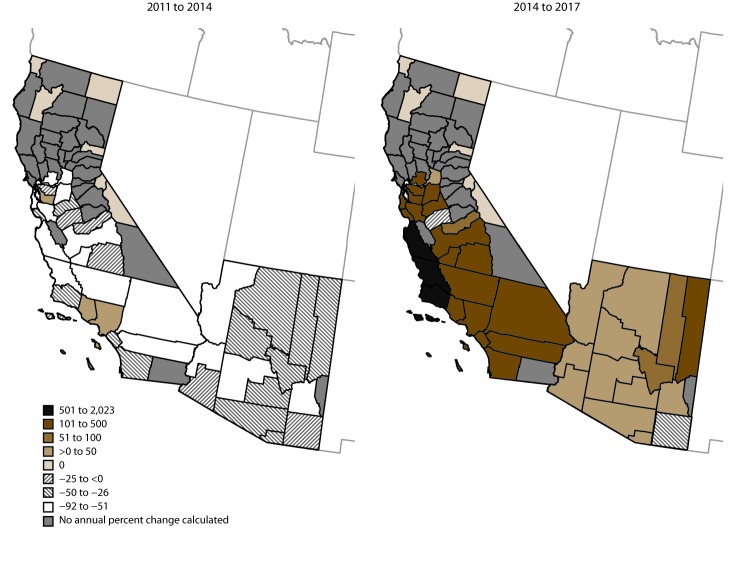
Annual percent change* in coccidioidomycosis incidence, by county — Arizona and California, 2011 to 2014 and 2014 to 2017 * Breaks were manually determined to best represent the range of the data shown. No annual percent change was calculated for counties that reported no cases in endpoint years or reported <25 cases overall during 2011–2017.

The number of cases decreased from 22,634 in 2011 to 8,232 in 2014 and subsequently increased to 14,364 in 2017. In Arizona, the incidence decreased from 260.5 cases per 100,000 population in 2011 to 90.8 in 2013 (average annual percent change [APC]: −41%) and was stable from 2013 to 2017 (Table 1). The average annual incidence was highest in Maricopa County (166.0 per 100,000 population), Pinal County (150.7), and Pima County (120.3) (Figure 2). The percent change in average annual incidence was negative in all Arizona counties during 2011–2014 and positive in all counties except Cochise during 2014–2017 (Figure 3).

**TABLE 1:** Coccidioidomycosis incidence,* by area — 26 states and the District of Columbia, 2011–2017

Year	Arizona	California	Nevada, New Mexico, and Utah combined	All other states combined^†^
2011	260.5	15.7	3.2	0.3
2012	202.3	12.1	2.8	0.3
2013	90.8	8.9	2.1	0.2
2014	85.8	6.0	2.0	0.3
2015	114.8	8.1	2.6	0.3
2016	90.5	14.2	1.9	0.3
2017	101.0	18.2	3.5	0.4

* Cases per 100,000 population, calculated using intercensal estimates from the U.S. Census Bureau.

^†^ All other states refers to all other states where coccidioidomycosis was reportable.

In California, the incidence decreased from 15.7 per 100,000 population in 2011 to 6.0 in 2014 (APC: −27%) and increased to 18.2 in 2017 (APC: 48%). Kern County (217.1), Kings County (138.6), San Luis Obispo County (59.1), and Fresno County (50.6) had the highest average annual incidence. The percent change in average annual incidence from 2014 to 2017 was highest in counties in the Central Coast (Figure 3).

The incidence in Nevada, New Mexico, and Utah combined decreased from 3.2 in 2011 to 2.0 in 2014 (APC: −15%); in 2017, the incidence was 3.5 (not significantly different from 2014). In all other states combined, the incidence was relatively stable throughout the surveillance period (average annual incidence: 0.3 per 100,000 population).

### Demographics

**FIGURE 4:**
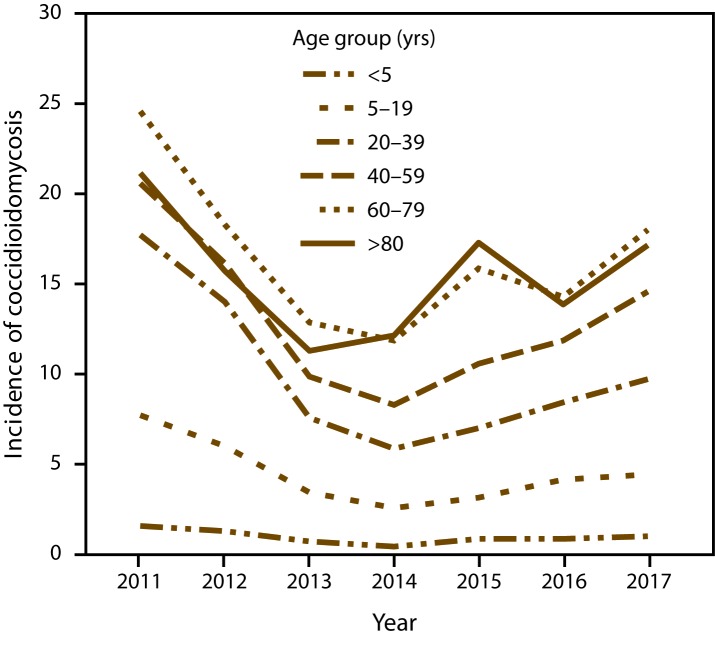
Annual incidence* of coccidioidomycosis, by age group — 26 states and the District of Columbia, 2011–2017 * Per 100,000 population, calculated using intercensal estimates from the U.S. Census Bureau.

Most cases occurred in adults aged 40–59 years (33.8%) and ≥60 years (31.6%) (Table 2). Nationwide, adults aged ≥60 years consistently had the highest incidence over time (Figure 4). The median age in California (45 years, interquartile range [IQR]: 30–58 years) was lower than in Arizona (51 years; IQR: 34–66 years); Nevada, New Mexico, and Utah combined (56 years; IQR: 42–68 years); and other states combined (62 years; IQR: 48–71 years).

**TABLE 2:** Demographic characteristics of patients with coccidioidomycosis and event date type, by area — 26 states and the District of Columbia, 2011–2017

Characteristic	Arizona	California	Nevada, New Mexico, and Utah combined	All other states combined*	Total
	N = 61,480	N = 30,979	N = 1,394	N = 1,518	N = 95,371
	No. (%)	No. (%)	No. (%)	No. (%)	No. (%)
**Median age, yrs (range)**	51 (0–107)	45 (0–103)	56 (5–96)	62 (7–94)	**49 (0–107)**
**Age group, yrs (n = 94,967)**					
<5	248 (0.4)	239 (0.8)	0 (0.0)	0 (0.0)	**487 (0.5)**
5–19	4,832 (7.9)	2,870 (9.3)	49 (3.5)	20 (1.3)	**7,771 (8.2)**
20–39	14,728 (24.1)	9,410 (30.5)	252 (18.1)	202 (13.4)	**24,592 (25.9)**
40–59	19,779 (32.3)	11,404 (37.0)	489 (35.1)	425 (28.3)	**32,097 (33.8)**
60–79	17,554 (28.7)	5,888 (19.1)	522 (37.4)	733 (48.7)	**24,697 (26.0)**
>80	4,087 (6.7)	1,030 (3.3)	82 (5.9)	124 (8.2)	**5,323 (5.6)**
**Sex (n = 94,882)**					
Male	27,918 (45.7)	20,122 (65.1)	861 (61.9)	922 (61.7)	**49,823 (52.5)**
Female	33,179 (54.3)	10,777 (34.9)	531 (8.1)	572 (38.3)	**45,059 (47.5)**
**Race (n = 31,787)**					
Native American/Alaska Native	972 (7.2)	106 (0.6)	23 (2.7)	9 (1.1)	**1,110 (3.5)**
Asian/Pacific Islander	431 (3.2)	1,332 (8.1)	54 (6.3)	18 (2.1)	**1,835 (5.8)**
Black	949 (7.0)	1,680 (10.2)	75 (8.7)	79 (9.3)	**2,783 (8.8)**
White	10,046 (74.2)	11,651 (70.5)	675 (78.4)	640 (75.6)	**23,012 (72.4)**
Other	1,146 (8.5)	1,766 (10.7)	34 (3.9)	101 (11.9)	**3,047 (9.6)**
**Ethnicity (n = 32,252)**					
Hispanic	2,121 (18.5)	8,728 (45.4)	168 (17.7)	56 (9.2)	**11,073 (34.3)**
Non-Hispanic	9,332 (81.5)	10,510 (54.6)	782 (82.3)	555 (90.8)	**21,179 (65.7)**
**Event date type (n = 95,221)**					
Onset date	3,846 (6.3)	11,275 (36.4)	619 (44.4)	425 (31.1)	**16,165 (17.0)**
Diagnosis date	1,115 (1.8)	8,587 (27.7)	543 (39.0)	127 (9.3)	**10,372 (10.9)**
Lab test date	31,000 (50.4)	9,029 (29.1)	170 (12.2)	551 (40.3)	**40,750 (42.8)**
Reported to county	204 (0.3)	1,955 (6.3)	32 (2.3)	167 (12.2)	**2,358 (2.5)**
Reported to state or CDC	25,315 (41.2)	133 (0.4)	30 (2.2)	98 (7.2)	**25,576 (26.9)**

* All other states refers to all other states where coccidioidomycosis was reportable.

**FIGURE 5:**
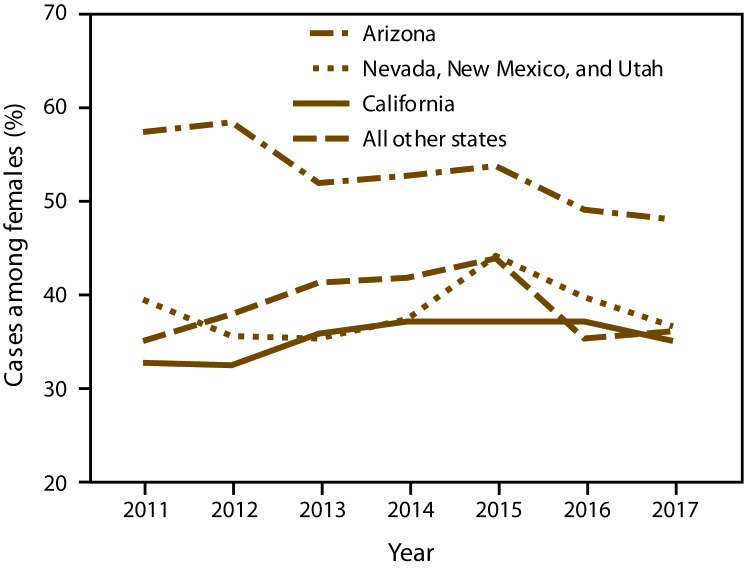
Proportion of coccidioidomycosis cases in females, by year and area* — 26 states and the District of Columbia, 2011–2017 * All other states refers to all other states where coccidioidomycosis was reportable.

Overall, more cases occurred in males (49,823; 52.5%); however, the distribution among males and females differed by area. In Arizona, 58.3% of cases were in females in 2012 but decreased to 48.1% in 2017. In contrast, in California; Nevada, New Mexico, and Utah combined; and other states combined, the predominance of cases in males stayed relatively consistent over time (Figure 5).

Information on race was available for 31,787 (33.3%) cases, although completeness varied by area, ranging from 22.0% in Arizona to 61.8% in Nevada, New Mexico, and Utah. Overall incidence per 100,000 persons was nearly twice as high in blacks compared with whites in Arizona (42.5 versus 25.4), California (9.5 versus 5.9), and Nevada, New Mexico, and Utah (3.0 versus 1.5) (Table 3). Information on ethnicity was available for 32,252 (33.8%) cases, ranging from 18.6% in Arizona to 68.1% in Nevada, New Mexico, and Utah. Incidence was higher among Hispanics than non-Hispanics in California (8.6 versus 6.6) but not in other areas (Table 3). Annual trends in race/ethnicity-specific incidence were similar to the overall incidence trends.

**TABLE 3:** Coccidioidomycosis incidence,* by race/ethnicity and area — 26 states and the District of Columbia, 2011–2017

Race/Ethnicity	Arizona	California	Nevada, New Mexico, and Utah combined	All other states combined^†^
**Race**				
American Indian/Alaska Native	38.8	2.4	1.1	0.2
Asian/Pacific Islander	25.7	3.3	2.0	0.1
Black	42.5	9.5	3.0	0.1
White	25.4	5.9	1.5	0.1
**Ethnicity**				
Hispanic	15.1	8.6	1.1	0.2
Non-Hispanic	29.3	6.6	2.0	0.1

* Cases per 100,000 population, calculated using intercensal estimates from the U.S. Census Bureau.

^†^ All other states refers to all other states where coccidioidomycosis was reportable.

### Seasonality

**FIGURE 6:**
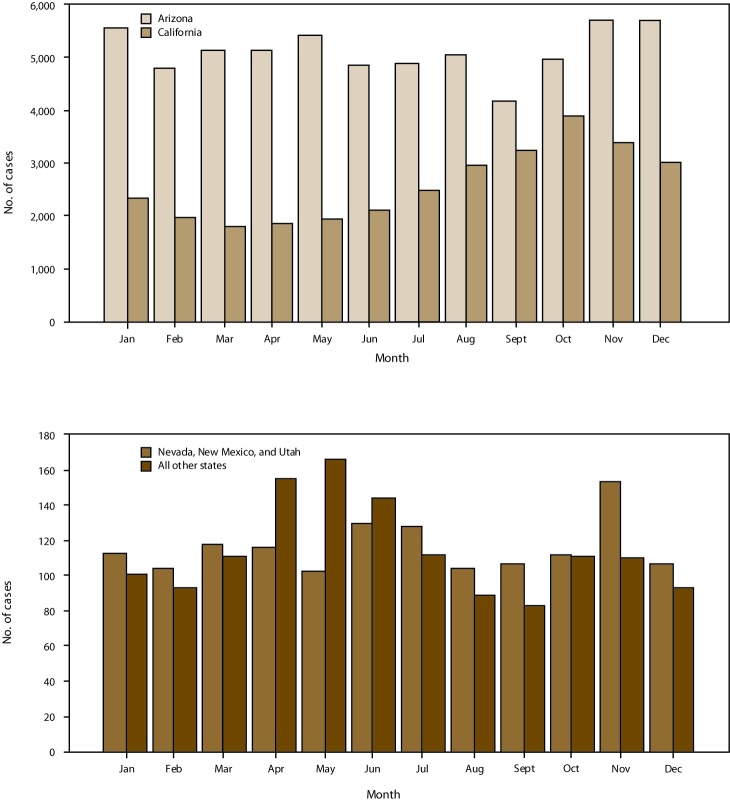
Number of coccidioidomycosis cases, by event month* and area^†^ — 26 states and the District of Columbia, 2011–2017 * Event month represented the earliest date associated with the case, which could be the symptom onset date, diagnosis date, laboratory test date, or the month reported to the county or state health department. ^†^ All other states refers to all other states where coccidioidomycosis was reportable.

Information on the type of event date was available for 95,221 cases (99.8%). The most common event date types were onset date in California (11,275 cases [36.4%]) and Nevada, New Mexico, and Utah (619 [44.4%]) and laboratory test date in Arizona (31,000 cases [50.4%]) and other states (551 [40.3%]) (Table 1). By event month, the number of cases peaked in the fall in California and had a bimodal distribution in other areas, with peaks in winter and spring (Figure 6). These trends also were apparent when the analysis was limited to cases with onset date as event date.

## Discussion

This report summarizes the incidence, demographic characteristics, and seasonality of 95,371 coccidioidomycosis cases reported to CDC from 26 states and DC during 2011–2017. The number of reported cases indicates that coccidioidomycosis remains an important public health problem both in the western United States and nationwide. For the first time since national coccidioidomycosis surveillance began in 1995, California reported more cases than Arizona in 2017.

Reasons for the overall decrease and recent subsequent increase in reported cases are not entirely clear but could involve weather or environmental factors, surveillance artifacts due to changes in testing or reporting practices, or changes in the susceptible population. Two known changes in testing and reporting could have influenced the trends that occurred during this surveillance period. First, California implemented mandatory laboratory reporting for several reportable conditions in 2010, which could have contributed to increased coccidioidomycosis case counts beginning in 2011 (*8*). However, this does not explain the subsequent decrease from 2011 to 2014, particularly because some high-incidence counties were already using laboratory-based reporting before 2010 (*16*). Second, a major commercial laboratory that typically reports >70% of the coccidioidomycosis cases in Arizona began using a different test kit in late 2012, leading to a decrease in cases reported by positive enzyme immunoassay alone the following year (*4*,*13*). However, cases also decreased substantially in all other areas of the United States from 2012 to 2013, suggesting that other factors also were involved in this decrease. Similarly, coccidioidomycosis hospitalization trends in both Arizona and California reported elsewhere (*13*,*17*) are similar to trends for cases reported to NNDSS, suggesting that changes in the environment or the population also influenced the observed trends.

Environmental factors such as temperature, precipitation, and wind affect the growth and dispersal of *Coccidioides* organisms. After several years of drought, increased rainfall in California in early 2016 might have resulted in more favorable conditions for *Coccidioides* and, consequently, more infections (*4*,*9*). Preliminary data from Arizona show an increase in cases during October 2017–March 2018, which could be partly related to particularly warm and dry weather during 2017 (*10*). In addition, Maricopa County experienced the largest population growth of any U.S. county during 2016–2017, which likely included persons who are immune-naïve to coccidioidomycosis (*10*).

At the county level, areas with high incidence were typically consistent with known high geographic risk areas in southern Arizona and California’s southern Central Valley. In addition, high average annual percent change in incidence also occurred outside of these areas, in east-central Arizona and California’s Central Coast, as previously described in 2016 (*9*). The reasons for the apparent increase in coccidioidomycosis in these areas are unknown and require further investigation but indicate that clinicians and the public should continue to be aware of the risk for coccidioidomycosis outside of the areas where the disease is traditionally highly endemic.

Age patterns in Arizona and California were consistent with previous reports (*4*,*8*,*13*,*18*). The higher median age in states outside of these areas suggests that cases occur in persons who travel regularly to or spend part of the year living in Arizona; these seasonal residents are typically older, retired adults (*6*). The overall predominance of cases in males also is consistent with previous reports (*4*,*8*,*13*,*18*). In Arizona, a higher proportion of cases in females first occurred in 2009 and lasted until 2016. These patterns are likely related to unknown changes in testing or reporting practices rather than environmental changes (*4*,*8*,*13*), especially because the same trends were not observed among cases reported from states where the disease is not endemic because many of these persons were probably exposed in Arizona. Another possible explanation involves differences in awareness or care-seeking behaviors. Enhanced surveillance in Arizona indicated that persons who knew about coccidioidomycosis before seeking health care were diagnosed more quickly than those who did not know about the disease (*14*). A recent analysis of Arizona Behavioral Risk Factor Surveillance System data indicated that women were more likely than men to know someone who had coccidioidomycosis (*19*).

The race/ethnicity findings in this report should be interpreted with caution because of the large amount of data that are missing. Arizona had the most missing data, which is not surprising because of statewide use of a laboratory-only case definition. Although limited, these race/ethnicity data are worthwhile to analyze, and they support some of the well-known associations between coccidioidomycosis and race/ethnicity, namely, that blacks and Filipinos are at higher risk for developing severe or disseminated disease. Some evidence also suggests that blacks, Filipinos, and Hispanics are at higher risk for acquiring coccidioidomycosis in general (*2*). This finding underscores the importance of analyzing differences in race/ethnicity-specific coccidioidomycosis rates by geography because the demographic composition of areas where the disease is highly endemic differs from that of the United States as a whole (*20*,*21*). Understanding coccidioidomycosis-related racial/ethnic disparities is important for developing targeted awareness and testing messages because certain groups at higher risk for severe disease might be less likely to correctly identity coccidioidomycosis symptoms (*19*).

The seasonal patterns in event month are similar to those observed in state-specific reports (*13*,*18*), with a fall peak in California and winter and spring peaks in Arizona and states outside the areas where coccidioidomycosis is endemic (probably reflective of cases acquired during travel to Arizona). Importantly, these peaks might not correspond with periods of increased exposures because of delays between exposure, symptom onset, seeking health care, being tested for and receiving a diagnosis of coccidioidomycosis, and reporting to public health authorities (*13*,*14*).

Coccidioidomycosis causes severe and prolonged illness in some patients; however, national surveillance does not collect information related to severity or outcomes such as body sites affected, hospitalization or immunocompromised status, antifungal treatment, or death. Routinely collecting this information for all cases would be extremely resource-intensive for states and counties that report thousands of cases each year and might not always lead to a better understanding of the disease in those areas. However, collecting such data on a sample of reported cases each year or performing periodic or geographically focused enhanced surveillance could help identify newly emerging high-risk populations, settings, or activities. Collecting enhanced data is also beneficial in areas where coccidioidomycosis appears to be newly emerging, such as the Pacific Northwest. These data can help public health investigators understand whether cases are travel associated or locally acquired, particularly when coupled with sequencing data from a clinical isolate (*4*). In states where *Coccidioides* is not present in the environment, detailed epidemiologic follow-up can help distinguish between coccidioidomycosis and histoplasmosis, blastomycosis, or other fungal diseases that are more common in those areas, and cross-reactivity can cause false-positive coccidioidomycosis serologic test results (*6*).

Current coccidioidomycosis prevention efforts focus on secondary prevention, aimed at improving outcomes among infected persons through greater clinical and public awareness, increased and earlier diagnostic testing, and earlier administration of antifungal treatment when necessary. Continued evidence of delays in seeking care and receiving diagnoses (*6*,*14*) and of frequent misdiagnoses suggests that increased prevention activities are warranted.

Primary prevention of coccidioidomycosis is challenging because of the limited understanding of the distribution of *Coccidioides* spp. in the environment, geographic and temporal variation in human exposures, and the relation between exposure dose and illness. Outbreaks, which have been detected primarily in occupational or institutional settings, provide some of the only data on specific risky exposures and primary prevention strategies (*22*). These strategies have included use of engineering controls, such as environmental modification (e.g., wetting soil before digging), and personal protective equipment (N-95 respirator or higher) during construction work in areas where the disease is endemic and excluding certain high-risk groups from incarceration in areas where the disease is highly endemic (*23*).

## Limitations

The findings in this report are subject to at least two limitations. First, the number of coccidioidomycosis cases reported to NNDSS is likely an underestimate of the actual number of cases that occur nationwide because of underdiagnosis and underreporting. Some persons infected with *Coccidioides* develop a relatively mild illness and either do not seek medical care or do seek care but never receive a diagnosis of coccidioidomycosis, partially because interpretation of test results is complex. Because coccidioidomycosis is only reportable in approximately half of states, many cases are likely undetected by public health practitioners. For example, although *Coccidioides* is known to be present in western Texas, coccidioidomycosis is not reportable in Texas except in the city of El Paso (*24*). Preliminary modeling estimates of the actual number of cases suggest that the number of symptomatic cases nationwide could be 6–14 times higher than the number reported to public health authorities (*25*). Nevertheless, NNDSS is one of the most comprehensive national data sources on coccidioidomycosis. Second, the case counts presented in this report might differ from those in previous state-specific reports. These discrepancies are due, in part, to reporting delays between the local and state level and between state health departments and NNDSS; some state health departments do not finalize their yearly case counts until after NNDSS finalizes case counts. For example, until recently, local health investigators in some California counties with high numbers of cases were either devoting substantial time and resources to investigating whether cases met the clinical criteria for the CSTE case definition or were reporting confirmed cases based on laboratory reports only. In January 2019, California began using a laboratory-based case definition, standardizing reporting practices across the state and likely reducing case-count discrepancies among public health agencies (*15*).

## Future Directions

Improving prevention and treatment of coccidioidomycosis relies on a better understanding of the epidemiology of the disease. This requires accurate, timely data that are comparable across public health jurisdictions. Strengthened coccidioidomycosis surveillance could involve revising the CSTE case definition to better reflect how cases are being reported; defining standardized data elements to systematically capture optional, more detailed information; and expanding surveillance to more states, particularly those with suitable habitats for *Coccidioides*. Research to determine the relative contributions of testing and reporting changes; environmental factors such as weather, climate, and land use; and population susceptibility would be useful for understanding how to interpret long-term surveillance data trends. Other areas for continued work include developing, evaluating, and promoting new methods for faster diagnosis and treatment. A new lateral flow assay can detect total antibodies against *Coccidioides* spp. in approximately 30 minutes but is not yet widely used (*26*). Research to determine the optimal treatment for uncomplicated pulmonary coccidioidomycosis is needed because whether antifungal medications improve symptom duration or intensity is unknown. Last, an effective vaccine could prevent substantial morbidity and mortality; a live-attenuated vaccine is being evaluated in dogs and might be effective in humans (*27*).

## Conclusion

This report provides an updated description of coccidioidomycosis epidemiology in the United States. During 2011–2017, the overall number of reported cases decreased and then subsequently increased, although different trends emerged in Arizona and California. The demographic features and seasonality of reported cases are consistent with previous reports. Despite the limited scope and depth of current coccidioidomycosis surveillance practices, these data indicate that the disease persists as an important national public health problem, with cases occurring across the country, and a major public health problem for parts of Arizona and California, where rates of reported cases in some counties exceed 100 per 100,000 population. Health care providers should consider a diagnosis of coccidioidomycosis in patients who live or work in or have traveled to areas with known geographic risk for *Coccidioides* and be aware that those areas might be broader than previously recognized. Ongoing public health efforts aimed at increasing awareness among the public and among health care providers are essential for helping patients receive a diagnosis and appropriate treatment more quickly. Research on understanding and reducing human exposures, individual susceptibility to disease, and vaccines could lead to more effective primary prevention strategies.
